# Meath Hospital

**Published:** 1832-10

**Authors:** 


### MEATH HOSPITAL.

### *Hysterical Vomiting and Neuralgia, cured by very large Doses of Assafœtida. Case reported by* Mr. DWYER.*

JULY 1832. Anne May, æt. twenty-nine, married, has had four children, her last, two years since, stillborn; after which confinement she got cold, with pain in the left side, shooting from the scapula to the region of the heart. She was admitted into this hospital three months ago, for a severe attack similar to the present, together with some fever, and was dismissed relieved, having been bled, leeched, and blistered. Admitted on the 5th July. She states that, for the last fortnight, she has suffered from pain shooting from the backbone, and along the course of the ribs till it arrives opposite the heart, when vomiting of bilious matter is induced by its severity. Never vomits without this precursory pain. At present, she rejects every thing from her stomach; no tenderness of any part of the abdomen on pressure; her general aspect is excited, and her respiration is extremely hurried, irregular, and accompanied by heaving of the chest and occasional sighing. This state of the respiration appears to persist during the whole period of the attack, which, however, in its other symptoms is variable, and consists of paroxysms, alternating with intervals comparatively calm. She lies for some time quiet on her back, and then suddenly starts up, rolls about in the bed, shrieking with agony, weeping, and agitated by violent eructations and vomiting, without, however, any disturbance of the pulse. Has never had Globus hystericus, nor has she been subject to headach or pain in the temple; appetite, previous to this attack, pretty good. Catamenia always regular; bowels generally confined; urine scanty, and deposits a copious sediment; pulse sixty-four; tongue moist; complains of thirst, (perhaps from vomiting.)

On examination of the spine, she shrinks from pressure over the dorsal spines and along the projections of the ribs round to the left

* From a paper by Dr. GRAVES, in the Dublin Journal of Medical and Chemical Science.

mamma. No palpitation of heart; no morbid phenomenon detected by stethoscope.

6th. Ordered actual cautery, to six points on each side of dorsal spines; and Assafœtidæ gr. x. 2ndis horis.

7th. Paroxysms of pain and vomiting occurred frequently up to twelve o'clock last night, when they ceased, and have not since returned. The cautery was applied, and she took twenty-two pills. Bowels confined, urine scanty and thick; other functions natural. Some tenderness still; respiration now quite tranquil; slept well. —Enema fetidum bis die. Repet. pilulæ 3tiis horis.

8th. No return of pain or vomiting; there is still tenderness on pressure, but less in degree; slept well; took sixteen pills, and had the two fetid enemata, which produced two scanty evacuations of hard fæces; respiration and other functions natural; bad appetite, she does not care for food. Convalescent.

13th. To-day she has some wandering pains in the right side, not severe.

*Observations.* My experience in other cases of a similar nature enables me to attribute the cure of this to the assafœtida, and not to the cautery. It is worthy of attention that she had taken 120 grains of assafœtida before the disease yielded, and that the improvement was permanent. In hysteria, when the patient can be prevailed on to take this medicine, I know nothing more efficacious than assfœtida; but to be serviceable it must be given in very large doses, as has been long ago remarked by practical physicians. When exhibited in small doses, as is usually the case, it too frequently appears to be inert, and consequently has of late rather fallen into disrepute.

---

